# Identification of key genes and their correlation with immune infiltration in osteoarthritis using integrative bioinformatics approaches and machine-learning strategies

**DOI:** 10.1097/MD.0000000000035355

**Published:** 2023-11-17

**Authors:** Duo Xia, Jing Wang, Shu Yang, Cancai Jiang, Jun Yao

**Affiliations:** a Department of Bone and Joint Surgery, The First Affiliated Hospital of Guangxi Medical University, Nanning, Guangxi, China; b Joint Surgery and Sport Medicine Department, Hunan Provincial People’s Hospital (The First Affiliated Hospital of Hunan Normal University), Changsha, Hunan Province, People’s Republic of China.

**Keywords:** biomarkers, GEO, LASSO, osteoarthritis, RandomForest, SVM-REF

## Abstract

Osteoarthritis (OA) is a common degenerative joint disease and is closely associated with chronic, low-grade inflammation. Regulating ferroptosis by targeting ferroptosis-related genes may be a fast and effective way to delay the degeneration of OA. However, the molecular mechanisms and gene targets related to ferroptosis in OA are still unclear. Data of OA samples from 3 gene expression omnibus (GEO) datasets were combined to identify differentially expressed genes (DEGs). Ferroptosis-related genes (FRGs) retrieved by the Ferroptosis database were intersected with DEGs, and the intersected hub genes were used for functional enrichment analysis. The feature genes were obtained from the least absolute shrinkage and selection operator (LASSO) algorithm, support vector machine recursive feature elimination (SVM-RFE) algorithm, and random forest (RF) algorithm. Single sample gene set enrichment analysis (ssGSEA) was used to compare immune infiltration between OA patients and normal controls, and the correlation between feature genes and immune cells was analyzed. The expression levels of feature genes were confirmed by RT-PCR. In addition, to explore the applicability of these genes, we extended the bioinformatics analysis of these feature genes to cancer. Finally, 4 feature genes, GABARAPL1, TNFAIP3, ARNTL, and JUN, were confirmed in OA. Theirs expression level were validated by RT-PCR. ROC curves of the 4 genes exhibit excellent diagnostic efficiency for OA, suggesting that the 4 genes were associated with the pathogenesis of OA. Another GEO dataset validated this result. Further analysis revealed that the 4 feature genes were all closely related to the immune infiltration cells in OA. Additionally, results of prognosis analysis indicated that JUN might be a promising therapeutic target for cancer. GABARAPL1, TNFAIP3, ARNTL, and JUN may be predicted biomarkers for OA. The feature genes and association between feature genes and immune infiltration may provide potential biomarkers for OA prediction along with the better assessment of the disease.

## 1. Introduction

Osteoarthritis (OA) is the most common type of chronic arthritis.^[1]^ With the advent of an aging society, the incidence of OA continues to rise, bringing great inconvenience to people lives and increasing social and economic burdens.^[2]^ At present, the molecular mechanism of OA is still unclear. Therefore, it is very important to deeply explore its pathogenesis and to prevent and treat osteoarthritis in the early stage.

Distinct forms of cell death are implicated in the etiopathology of chronic degenerative diseases.^[3]^ Ferroptosis may be one of the most common and oldest forms of cell death, and was involved in the progression of OA.^[4,5]^ The use of the ferroptosis inhibitor ferrostatin-1 can regulate the expression of type II collagen, relieve the degeneration of articular cartilage, and delay the progression of OA.^[6]^ Although the mechanism of ferroptosis in OA is unclear, targeting ferroptosis-related genes or using iron chelators might effectively inhibit ferroptosis.^[7]^ The study by Guo et al^[8]^ also confirmed that the use of iron chelator deferoxamine can inhibit chondrocyte ferroptosis and promote the activation of nuclear factor erythroid-2 related factor 2 (Nrf2) antioxidant system, effectively protecting cartilage cells. Targeting ferroptosis-related genes to regulate ferroptosis may be a fast and effective way to delay the degeneration of OA, but the molecular mechanisms and gene targets related to ferroptosis in OA have not been well-elucidated.

Bioinformatics can efficiently and intuitively describe the molecular mechanism of disease at multiple aspects, providing a large amount of effective information for basic medical research. The ferroptosis-related analysis and the establishment of biomarkers for OA-related biological information may provide an effective therapeutic potential for OA.

Machine learning (ML), encompassing methods such as least absolute shrinkage and selection operator (LASSO), support vector machine recursive feature elimination (SVM-RFE), and random forest (RF), and serving as an integral facet of artificial intelligence (AI), has seen a considerable expansion in its role within the healthcare system.^[9]^ The incorporation of ML not only augments the efficiency of various health care processes but also furnishes a more sophisticated and reliable methodology for exploring potential biomarkers. In contrast to conventional methods, the utilization of ML instills greater confidence in the scientific investigation, enabling more precise and insightful analyses.^[10]^

In this study, by integrating bioinformatics approaches and 3 ML strategies, the ferroptosis-related OA maker genes were screened out, and gene expression and ROC curve validation were performed in independent external datasets to provide an effective reference for the treatment of OA from the aspect of ferroptosis. A deeper exploration into the ferroptosis–related biomarker genes that expressing abnormally and their impact of immune infiltration will advance our understanding of the disease progression and mechanism in OA.

## 2. Materials and methods

### 2.1. Overview of research procedures

In the present study, OA-related gene chip datasets were accessed from Gene expression omnibus (GEO-https://www.ncbi.nlm.nih.gov/geo/) open resources. Genes expressing differentially between OA tissues and normal tissue samples were identified in data from GEO database. Three popular machine learning algorithms, LASSO, SVM-RFE, and RF classifier were employed to identify the most critical feature genes for further study. Furthermore, correlation analysis of selected feature genes and the abundance of immune cells was performed.

### 2.2. Data processing and download of the OA datasets

This study involved 10 OA cases and 9 control samples cases from GSE12021, 10 OA samples and 10 normal tissue samples from GSE55457 as well as 10 pairs of OA tissues and control non-OA samples from GSE55235, all the information of above datasets were obtained from the GEO database^[11]^ and were listed in Table 1. Batch effects of various GEO datasets were corrected using ComBat function from the R package “sva.”^[12]^ The 3 datasets were then consolidated as a larger cohort containing the gene expression matrix from 30 OA samples and 29 control non-OA samples. GSE1919 cohort based on GPL91 which consisted of 5 OA samples and 5 control samples were obtained for the validation. The Ferroptosis Database (FerrDb-http://www.zhounan.org/ferrdb/current/) was utilized for the ferroptosis information collection of ferroptosis-related genes (FRGs). The differences in mRNA expression are determined by “limma” package (http://www.bioconductor.org/packages/release/bioc/html/limma.html). The adjusted *P* values were adopted and examined in merged dataset to avoid the occurrence of false-positive results.

**Table 1:** Descriptive statistics.

Data number	Platform information	Osteoarthritis group	Control group	Control group
GSE12021	GPL96	10	9	Homo sapien
GSE55235	GPL96	10	10	Homo sapien
GSE55457	GPL96	10	10	Homo sapien

### 2.3. Identification of DEGs

We used the R “limma” package^[13]^ to screen DEGs between OA samples and control samples. The corresponding *P* value of the gene symbols after t test were used, and adjusted *P* < 0:05 and |log2FC|>1 were used as the selection criteria. Those with log2FC > 0 was identified as up-regulated expressed genes and those with log2FC < 0 was considered to be down-regulated one. The “pheatmap” and “ggplot2” packages^[14]^ in R were respectively employed to visualize the heatmap and volcano plots for DEGs.

### 2.4. Functional enrichment analysis

Gene ontology (GO) terms and kyoto encyclopedia of genes and genomes (KEGG) pathways analyses were conducted to annotate the functions of DEGs. The R packages used for this operation included “clusterProfile,” “enrichplot,” “ggplot2,” “org.Hs.e.g..db,” “GOplot,” and “DOSE.”^[15–17]^ The significance of enriched processes was determined using a cutoff of *P* value of .05. A term with *P* < .05 were regarded as significantly enriched. Besides, a Venn diagram was plotted and displayed the number of ferroptosis-related hub genes selected by the results of the 2 methods (DEGs and FRGs).

### 2.5. Screening of feature genes

Three ML methods were employed to identify the feature genes of OA. Using “glmnet” package in R,^[18]^ we performed LASSO regression to select the first part of feature genes. The tuning parameter (λ) of LASSO was selected by 10-fold cross validation method with minimum criteria.^[19]^ Using “e1071,” “kernlab,” and “caret” packages in R,^[20,21]^ we then applied another ML approach, SVM-RFE algorithm to identify the second subset of feature genes.^[22]^ The SVM-RFE algorithm removes the feature with the lowest score (least ranked) in each iteration and train remaining features again for next iteration. The whole process iterates until this algorithm finally select the second subset of feature genes. The selection of the third subset of feature genes were implemented with RF algorithm employing the “RandomForest” package^[23]^ in R. The intersection of the 3 subsets of feature genes selected by from the above 3 ML methods were taken and ultimately determined key feature genes.

### 2.6. Construction of receiver operating characteristic curves

Receiver operating characteristic (ROC) curves were plotted by using “pROC” function in the R package^[24,25]^ to estimate the diagnostic performance in OA. Validation of diagnostic role of feature genes for OA were conducted in GSE1919 dataset.

### 2.7. RT-PCR validation of the hub genes

To confirm the findings from the bioinformatics analysis, synovial tissue from 5 patients without OA and 5 patients with OA were harvested for RT-PCR validation. The study protocol was approved by the Ethics Committees of the First Affiliated Hospital of Guangxi Medical University, and all patients signed the informed consent. Total RNA from synovial tissue was extracted with TRIzol reagent (Invitrogen, Thermo Fisher Scientific, Inc.). RNA samples from total RNA were reverse transcribed to cDNA, and RT-PCR was carried out using the Revert Aid First Strand cDNA Synthesis Kit (Fermentas, USA). GAPDH was used as an internal reference. Relative mRNA expression was calculated using the 2-ΔΔCt method. One-way analysis of variance was used for the statistical analysis, and *P* < .05 indicated a significant difference.

### 2.8. Immune infiltration analysis by ssGSEA algorithm

Heterogeneity of immune cell content between OA tissues and normal tissues were analyzed with single sample gene set enrichment analysis (ssGSEA) method to explore the immune cell infiltrations in various samples.^[26]^ The correlation of different immune cell types and feature genes were investigated using the R “Corrplot” package.^[27]^ Spearman rank correlation test^[28]^ was utilized to statistically examine significance of relationships.

### 2.9. Survival analysis

A Venn diagram visualized the immune-related key gene obtained from the results of the 2 methods (feature genes and immune-related genes). We subsequently conducted the survival analysis of the immune-related key gene to explore the impact of the gene expression on the prognosis of patients with cancers. The primary survival outcomes we focused on basically included the overall survival (OS), disease-free survival (DFS)/ relapse-free survival (RFS), and progression-free survival (PFS). Taking the above 4 clinical outcomes as observations, we applied single stepwise regression analysis to investigated the hazard ratios (HR) of the expression of immune-related key gene. *P* value <.05 indicated a significant difference.

### 2.10. Immune cell infiltration analysis and immune checkpoints analysis

To explored the immune role at the pan-cancer level, we investigated the correlation of immune-related key gene expression and tumor-infiltrating lymphocytes (TILs), immunomodulators, and chemokines using TISIDB (http://cis.hku.hk/TISIDB/), an online tool helping analysis tumor-immune interactions.^[29]^

### 2.11. Statistical analysis

The software R (R version 4.1.2; https://www.r-project.org/) was used for statistical tests and data visualization in this study. The significance of correlation was analyzed by the Spearman correlation test. Indication of *P* value summaries were as follow: *P < .05, **P < .01, ***P < .001, and ****P < .0001.

## 3. Results

### 3.1. Identification of DEGs

**Figure 1.**
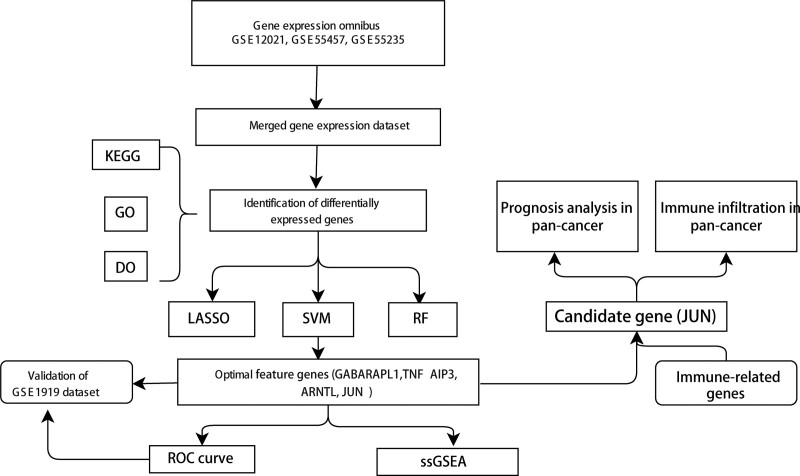
Flowchart of the study.

**Figure 2.**
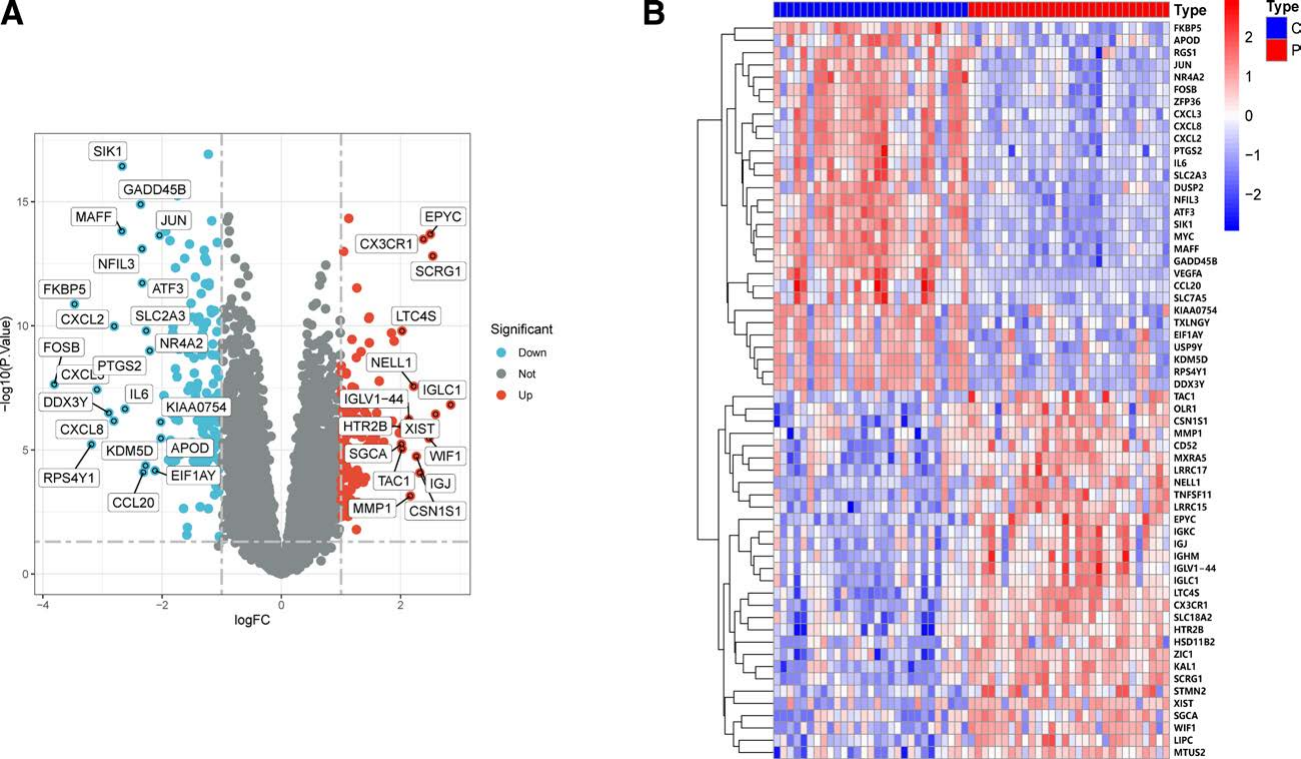
Differential expression analysis. (A) Volcano plot for DEGs; red dots represent upregulated differential genes, and the green dots represent downregulated differential genes (|log2FC|>1 and adjusted *P* < .05). (B) Cluster heatmap for DEGs in OA patients and normal controls. From blue to red represents the low expression to high expression. C = control, DEGs = differentially expressed genes, FC = fold change, OA = osteoarthritis.

A total of 259 FRGs were identified. The entire analysis process of this study was presented in the Figure 1. At first, we compared the genes expression levels of normal control and OA disease group to identify DEGs. When comparing the synovial tissues of 30 OA patients and 29 normal controls, we found that 126 genes were highly expressed in OA samples while 156 genes were down-regulated. The results of DEGs were shown in the volcano plot and heatmap (Figs. 2A and 2B).

### 3.2. Functional enrichment analysis

**Figure 3.**
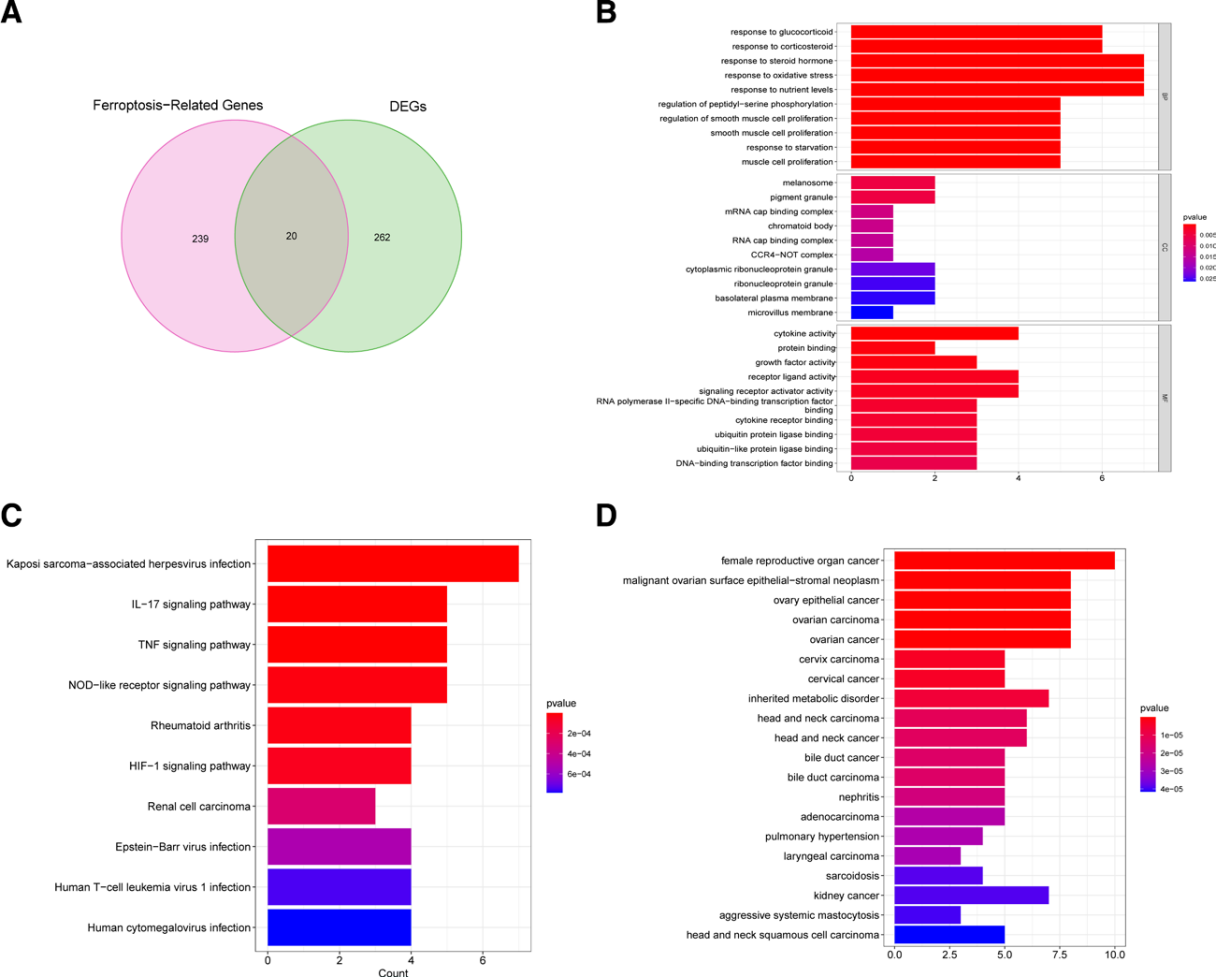
Functional analysis of key module genes merged with DEGs. (A) Venn diagram of key module genes versus DEGs. (B) DO analysis. (C) GO analysis. (D) KEGG analysis. DEGs = differentially expressed genes, KEGG = kyoto encyclopedia of genes and genomes.

We ultimately identified 20 ferroptosis-related hub genes by intersecting the obtained DEGs and 259 FRGs using the Venn diagram method (Fig. 3A). The top-ten enriched GO terms in biological processes (BP), cellular components (CC) and molecular functions (MF) were represented in Figure 3B. At the level of BP aspect, these DEGs were enriched in oxidative stress, steroid hormone, and muscle cell proliferation; for CC, these DEGs were significantly enriched in microvillus membrane, basolateral plasma membrane, and melanosome; and for MF, these DEGs were enriched in cytokine activity, protein binding, growth factor activity, and receptor ligand activity. KEGG pathway analysis was also performed and results shown that these DEGs were abundantly enriched in immune-related pathways, such as IL-7 signaling pathway, TNF signaling pathway, HIF-1 signaling pathway, Human T-cell leukemia virus 1 infection, and NOD-like receptor signaling pathway (Fig. 3C). Results of disease ontology (DO) analysis showed significant enrichment of DEGs in various cancers including ovarian cancer, cervical cancer, kidney cancer, and adenocarcinoma (Fig. 3D).

### 3.3. Selection of feature genes

**Figure 4.**
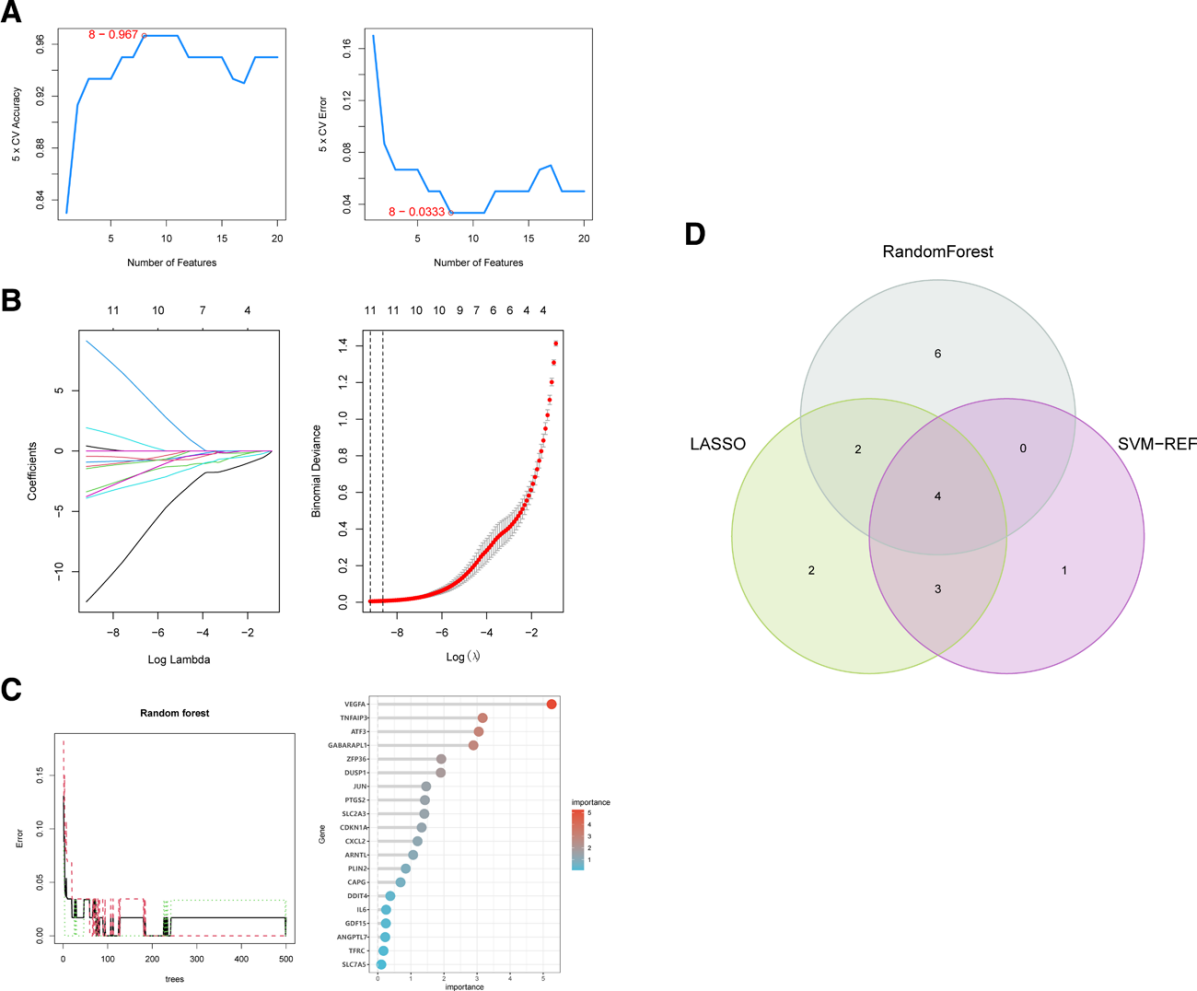
Feature gene selection. (A) Biomarker signature gene expression validation by support vector machine recursive feature elimination (SVM–RFE) algorithm selection. (B) Adjustment of feature selection in the minimum absolute shrinkage and selection operator model (lasso). (C) Random Forest error rate versus the number of classification trees. The top 20 relatively important genes were selected. (D) Three algorithmic Venn diagram screening genes.

**Figure 5.**
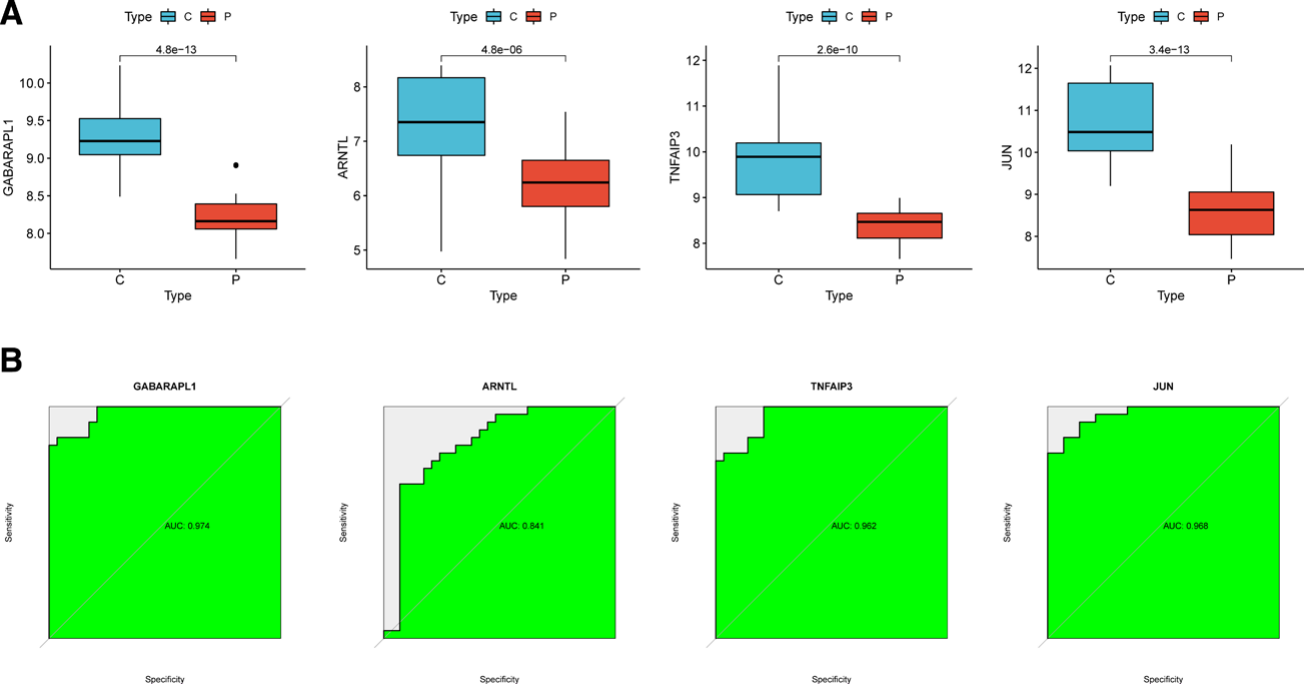
Gene expression of the feature genes and ROC curves. (A) Expression of GABARAPL1, TNFAIP3, ARNTL, and JUN in OA patients compared to normal controls in the merged dataset. (B) ROC curves of the predictive efficacy of GABARAPL1, TNFAIP3, ARNTL, and JUN in the merged set. AUC, area under the curve; ROC, receiver operating characteristic.

Three ML algorithms simultaneously employed for the identification of feature genes, SVM-RFE algorithm selected 8 genes from ferroptosis-related hub genes (Fig. 4A), LASSO algorithm identified eleven genes (Fig. 4B) and RF algorithm provided ten genes (Fig. 4C). Taking the intersection of the results of genes selection by the 3 ML methods, we ultimately obtained 4 feature genes: GABARAPL1, TNFAIP3, ARNTL, and JUN (Fig. 4D). Followed correlation analysis among the expression of the 4 genes all showed positive correlations, suggesting an underlying synergy of the biological impact of the 4 genes (Figure S1, http://links.lww.com/MD/K714). We then performed difference analysis in merge data and reconfirmed the down-regulation of expression of the 4 genes in OA (Fig. 5A). The area under curve (AUC) of ROC analysis was 0.974 for GABARAPL1, 0.841 for ARNTL, 0.962 for TNFAIP3, and 0.968 for JUN (Fig. 5B).

**Figure 6.**
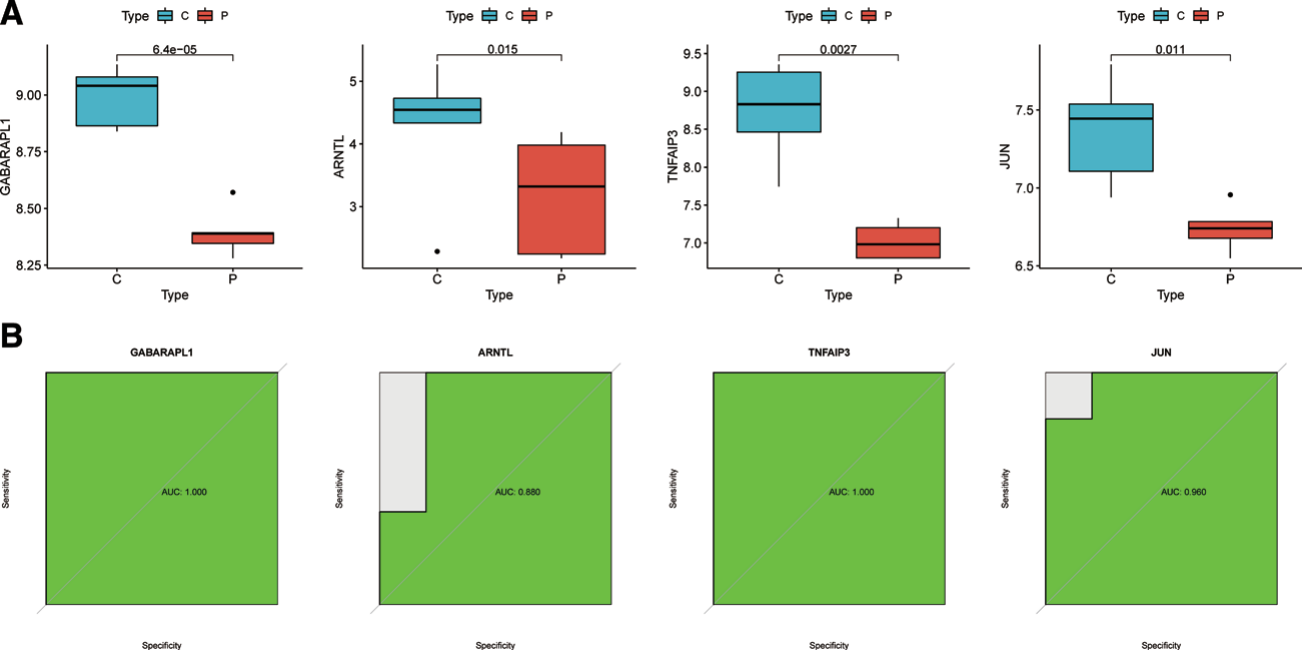
Validation of the feature genes and ROC curves. (A) Expression of GABARAPL1, TNFAIP3, ARNTL, and JUN in OA patients compared to normal controls in the validation dataset (only genes with *P* < .05 are shown). (B) ROC curves of the predictive efficacy of GABARAPL1, TNFAIP3, ARNTL, and JUN in the validation set.

The difference of the expression of the 4 feature genes between OA samples and corresponding normal tissue samples were confirmed again in the validation cohort (GSE1919 dataset). The expression levels of GABARAPL1, TNFAIP3, ARNTL, and JUN in OA samples were lower compared with that in the paired normal tissue samples (Fig. 6A). The results of ROC analysis (Fig. 6B) showed that AUC was 1.000 for GABARAPL1, 0.880 for ARNTL, 1.000 for TNFAIP3, and 0.960 for JUN.

### 3.4. RT-PCR validation of the feature genes

**Figure 7.**
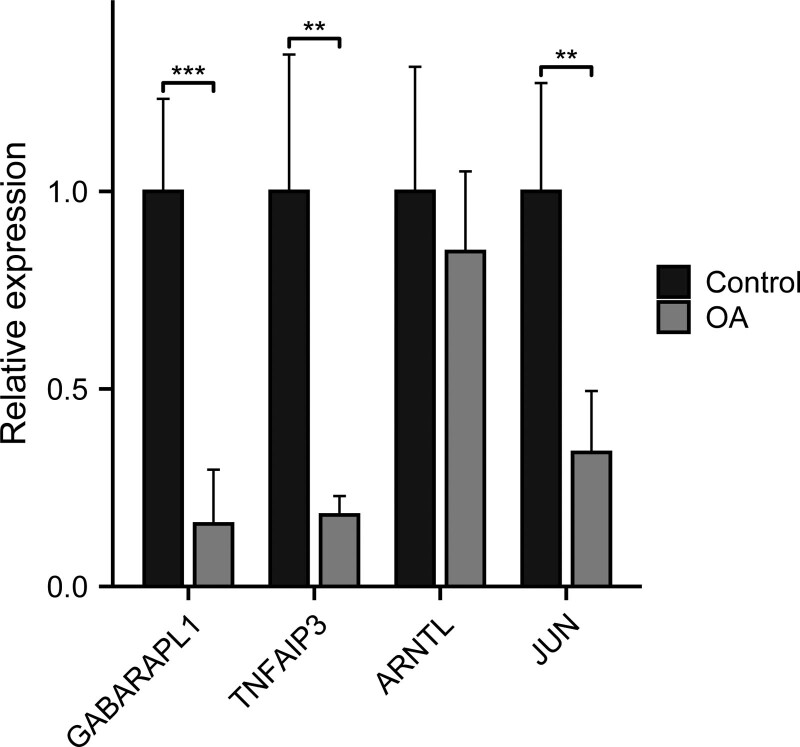
RT-PCR validation of the hub gene between OA and normal controls. All experiments were performed in triplicate and results were presented as M ± SD. (*P < 0:05, **P < 0:01, ***P < 0:001).

The results showed that the relative expression levels of 4 feature genes including GABARAPL1, TNFAIP3, and JUN were consistent with the results of bioinformatics analysis. ARNTL showed no statistically significant difference (Fig. 7).

### 3.5. Trait gene interaction analysis

A protein-protein interaction (PPI) network of the 4 feature genes was established using GeneMANIA database (Figure S2A, http://links.lww.com/MD/K715). GO and KEGG analysis of PPI genes were subsequently performed. Cellular response to oxidative stress, rhythmic process, and cellular response to extracellular stimulus were the most abundant BP terms. In CC category, PPI genes were mainly enriched in leading-edge membrane, transcription regulator complex, dendrite membrane, and cytoplasmic region. In the CC part, E-box binding, DNA-binding transcription factor binding, and MAP kinase activity were significantly enriched (Figure S2B, http://links.lww.com/MD/K715). The enriched KEGG pathways included rhythmic process, response to oxidative stress, and regulation of DNA-binding transcription factor activity (Figure S2C, http://links.lww.com/MD/K715).

### 3.6. Immunological infiltration in the OA group and healthy controls using ssGSEA analysis

**Figure 8.**
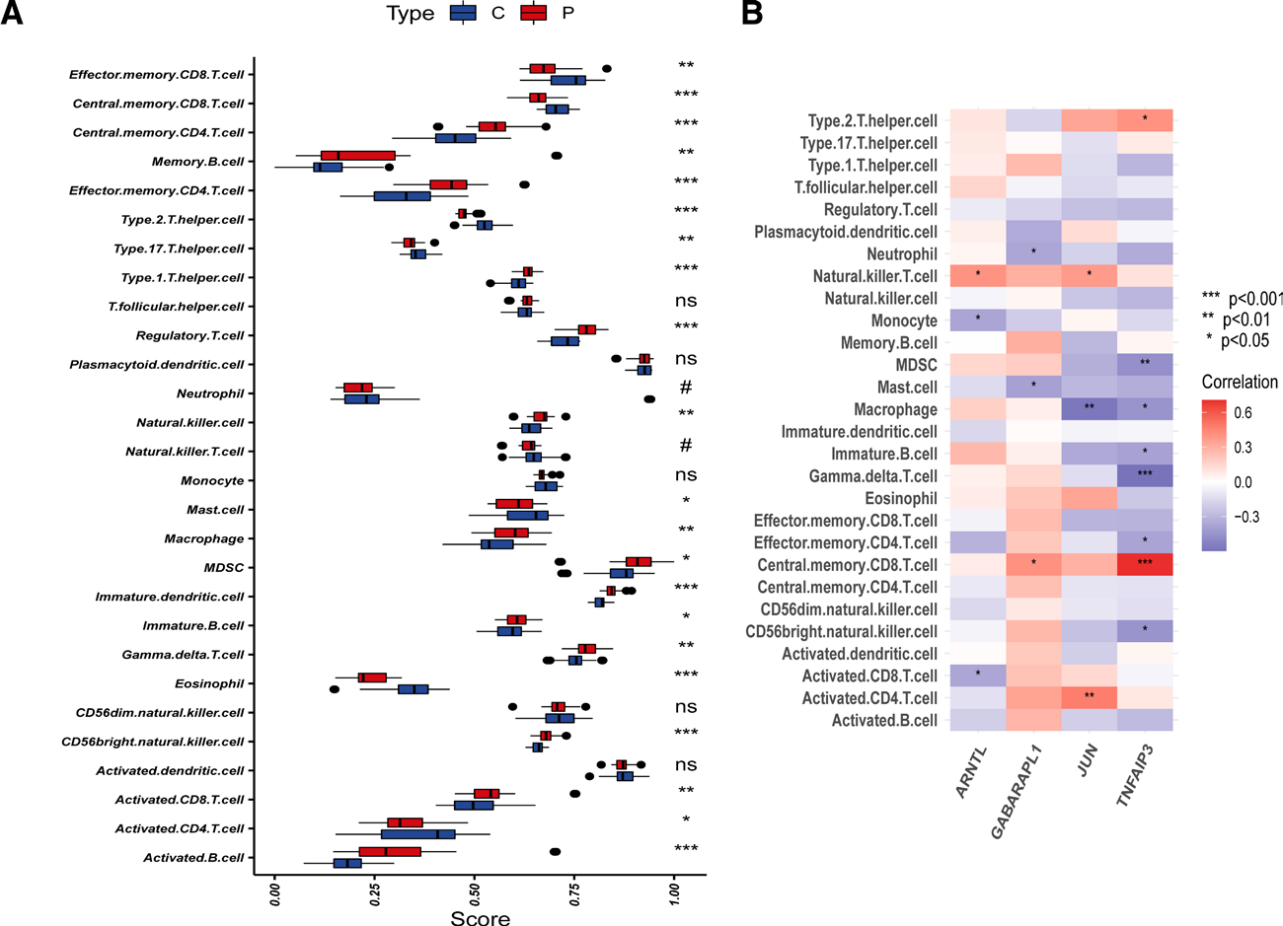
Correlation between OA and immunity. (A) Comparison of ssGSEA scores of immune cells and immune pathways between OA group and healthy controls. (B) Correlation between characteristic genes and immunity. *P < .05, **P < .01, ***P < .001. NS = no significance.

The results of ssGSEA showed that the proportion of Effector memory CD8 T cell, Central memory CD8 T cell, Type 17 T helper cell, Type 2 T helper cell, Mast cell, Eosinophil, and Activated CD4 T cell in OA samples were significantly lower than in normal control samples However, Central memory CD4 T cell, Memory B cell, Effector memory CD4 T cell, Type 1 T helper cell, Regulatory T cell, Natural killer cell, Macrophage, MDSC, Gamma delta T cell, Immature B cell, Immature dendritic cell, CD56 bright natural killer cell, Activated CD8 T cell, and Activated B cell in OA samples were significantly higher than in normal control samples (Fig. 8A). These results revealed that immune infiltration status had a significantly difference in OA samples, which advanced our comprehending of mechanism in OA. Correlation analysis shown that JUN was associated with Activated CD4 T cells, Macrophage, and Natural killer T cell (Fig. 8B). Our founding suggested that feature genes have an underlying impact on immune cells component.

### 3.7. Construction of a ceRNA network based on feature genes

**Figure 9.**
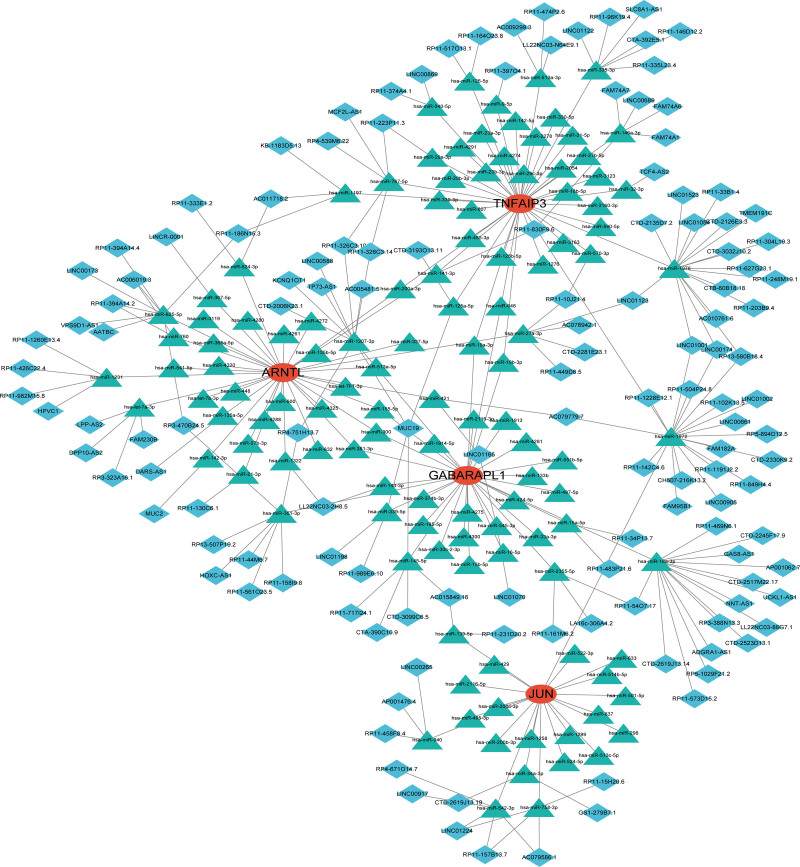
The ceRNA network based on 4 feature genes. The red color represents the hub genes, the green color denotes the predicted miRNA, and the blue color signifies the predicted lncRNAs.

Based on the interaction of the 4 feature genes with relevant miRNAs, lncRNAs by using miRanda (http://mirtoolsgallery.tech/mirtoolsgallery/node/1055), miRDB (https://mirdb.org/), TargetScan (https://www.targetscan.org/vert_72/), and spongeScan (https://spongescan.rc.ufl.edu/) databases, we constructed a regulatory network integrating multifactor including miRNA and lncRNA to explore the underlying regulatory mechanism of the expression of these feature genes at both transcriptional and post-transcriptional levels (Fig. 9).

### 3.8. Immune-related key gene expression

**Figure 10.**
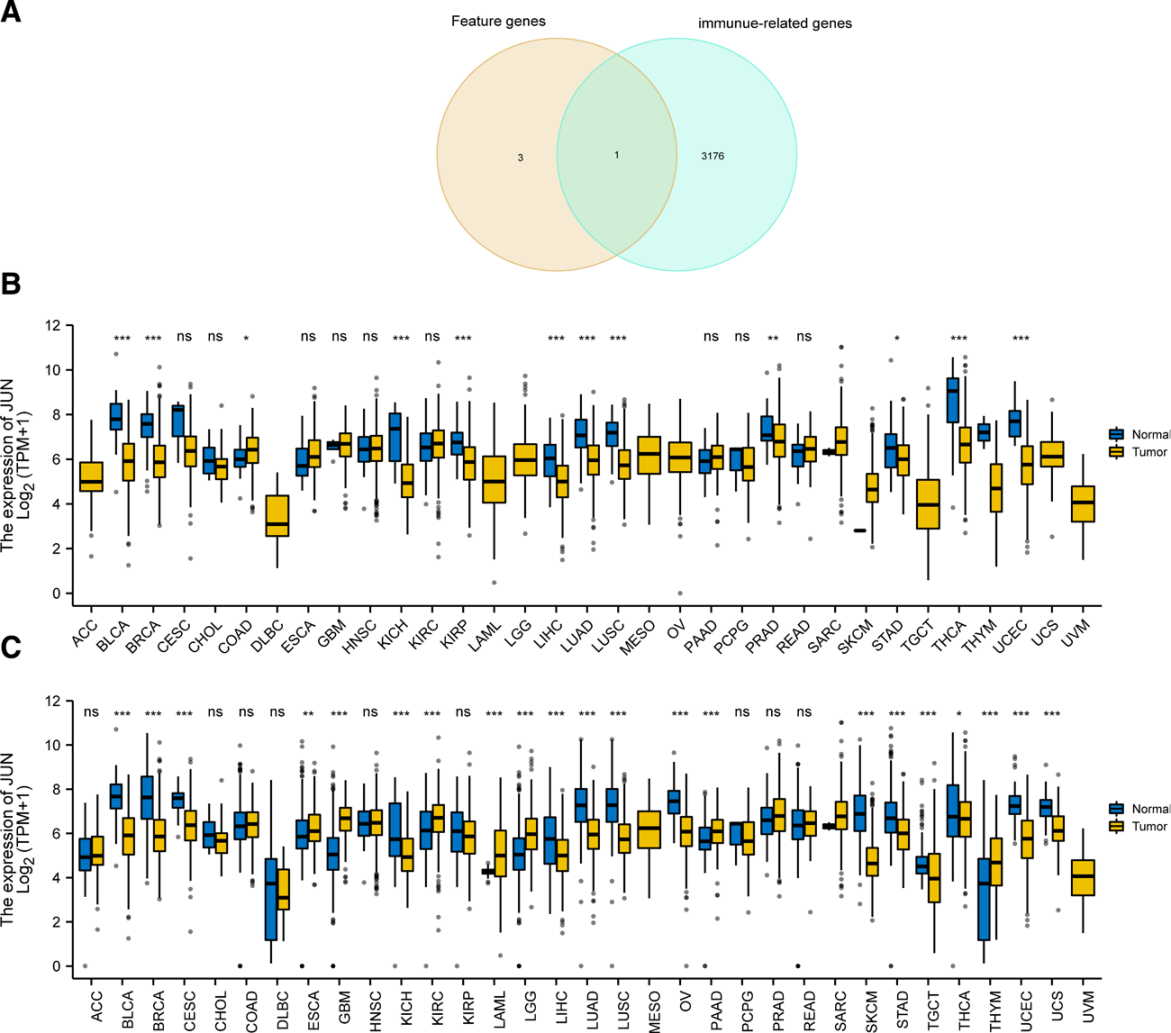
JUN expression. (A) Venn diagram between immune gene and hub genes. (B) Pan–cancer expression levels of JUN in the TCGA dataset. (C) Pan–cancer expression levels of JUN in the TCGA and GTEx datasets.

We then identified the intersection of the feature genes we obtained and immune-related genes included in 2 prior knowledge database InnateDB (https://www.innatedb.com/) and Immport (https://www.immport.org/home). Ultimately, JUN was the only genes met the requirements (Fig. 10A). Taking impact of JUN in immunity to account, we envisaged that JUN may play a role in anti-tumor immunity and turned our sights to the expression of JUN in various cancer. In subsequent pan-cancer analysis in TCGA database, down-regulation of JUN in BLCA, BRCA, KICH, KIRP, LIHC, LUAD, LUSC, PRAD, STAD, THCA, UCEC and up-regulated expression in COAD were observed (Fig. 10B). Another pan-cancer analysis performed in TCGA and GTEx databases and resultantly indicated that JUN expressed at low levels in a variety of cancer types including BLCA, BRCA, CESC, KICH, LIHC, LUAD, LUSC, OV, SKCM, STAD, TGCT, THCA, UCEC, and UCS, whereas highly expressed in ESCA, GBM, KIRC, LAML, LGG, PAAD, and THYM (Fig. 10C).

### 3.9. JUN prognostic value in pan-cancer

**Figure 11.**
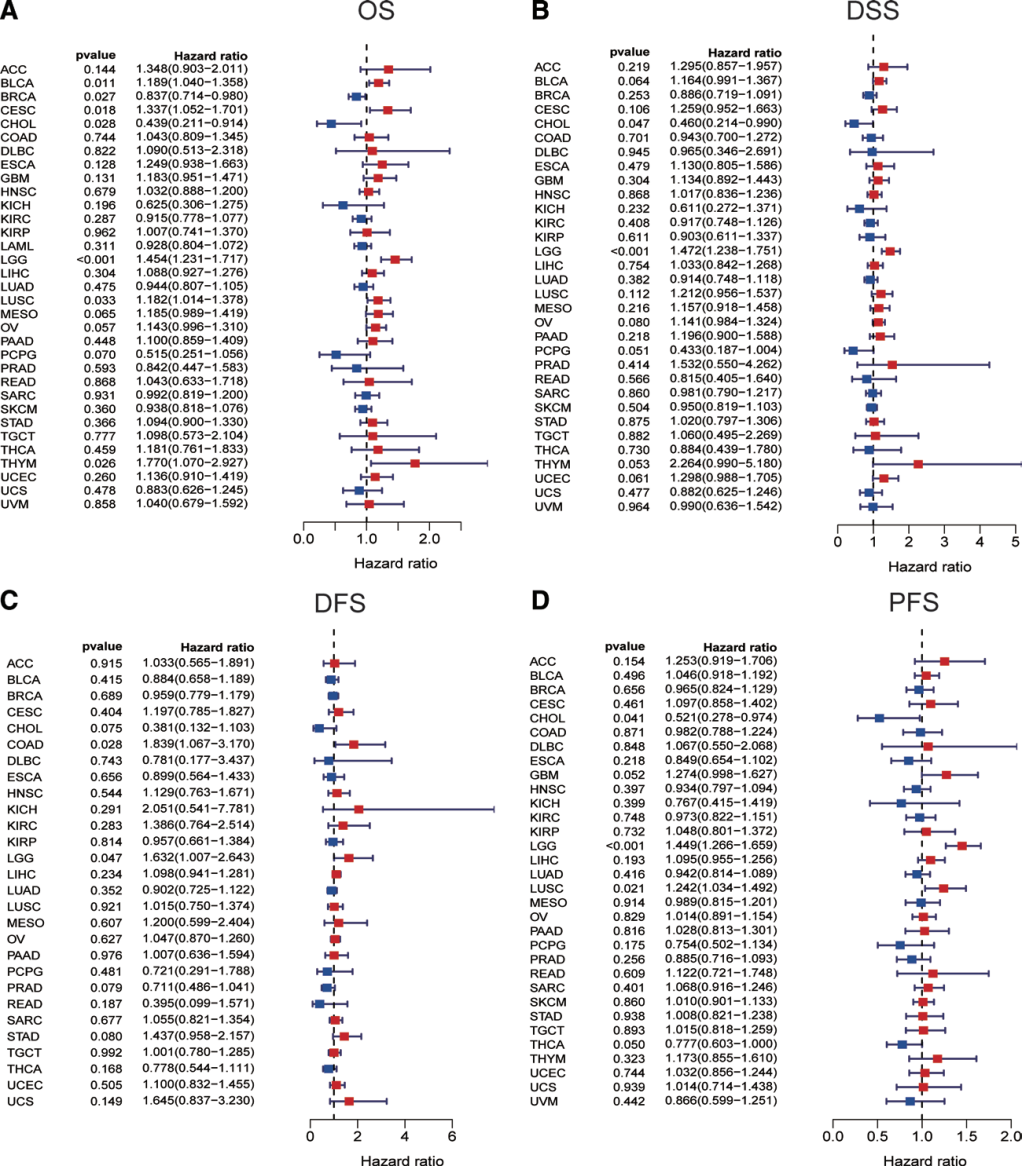
Correlation of JUN with prognosis in pan–cancer. (A) Cox regression model analysis of the correlation between JUN expression and OS in various tumors. (B) Cox regression model analysis of the correlation between JUN expression and DSS in various tumors. (C) Correlation analysis of JUN expression and DFS in various tumors by Cox regression model. (D) Correlation analysis of JUN expression and PFS in various tumors by Cox regression model.

We then investigated the correlation of JUN expression and the prognosis of patients with various cancer types. Taking OS as observation outcome, we found significant correlation between JUN expression and OS of 7 cancers: BLCA, BRCA, CESC, CHOL, LGG, LUSC, and THYM. (Fig. 11A). Cox regression of DSS of patients also showed that JUN expression was significantly associated with DSS of patients with CHOL and LGG. (Fig. 11B). Cox regression analysis of DFS of OA patients indicated that JUN expression had non-negligible impact of the DFS of COAD and LGG (Fig. 11C). In terms of PFS analysis, a significant correlation between JUN expression and the PFS of patients with CHOL, LGG, and LUSC (Fig. 11D). This finding demonstrated that JUN was closely associated with multiple cancer types, especially in LGG.

### 3.10. JUN expression was correlated to immune related molecules in pan-cancer

TILs, immunomodulators, and chemokines engaged in and affected immune function status of cancer, we further investigated the correlation of level of these immune response related-components and the JUN expression to gain a deeper insight on the role of JUN in pan-cancer using the TISIDB tool (http://cis.hku.hk/TISIDB/). We observed significant positive correlation among JUN expression and TILs, immunomodulators, and chemokines at pan-cancer level (Figure S3, http://links.lww.com/MD/K716). These results supported the findings that JUN may function as an immunoregulatory factor in pan-cancer, especially in LGG.

### 3.11. MSI and TMB analyses

Immunotherapies targeting PD-1/PD-L1 have made great clinical progress in immune checkpoint therapy.^[30–32]^ Elevated tumor mutation burden (TMB) or microsatellite instability (MSI) values have emerged as strong markers in predicting the immune checkpoint inhibitor (ICI) response.^[33–35]^ The association between the JUN expression and immunotherapy-related biomarkers (TMB and MSI) was further explored. The obtained results revealed that the JUN expression was positively associated with TMB in 11 cancer types (including BRCA, UCEC, THYM, THCA, STAD, PRAD, LUSC, LUAD, LGG, HNSC, and COAD) as well as MSI in 8 cancer types (including UCS, UCEC, THYM, TGCT, LUSC, LUAD, DLBC, and COAD) (Figure S4, http://links.lww.com/MD/K717). This finding suggest that JUN may be a potential indicator for immunotherapy response.

## 4. Discussion

Osteoarthritis, the most common chronic joint disease, has become a global health concern worldwide.^[36]^ As a degenerative joint disease, OA is generally characterized by the loss of articular cartilage, hypertrophic changes, abnormal osteophyte reformation, subchondral bone remodeling and synovitis. At present, the pathogenesis of the disease is still unclear, and drug treatment is mainly aimed at relieving symptoms and cannot effectively cut off or delay the development of OA. Currently, the main diagnostic methods of OA depend on physical examination, medical history and radiographic which remain relatively poor and late for the early diagnosis of OA. Thus, additional diagnostic tools are required for a definitive diagnosis of OA.^[37,38]^

Ferroptosis is one of forms of programmed cell death that involves iron accumulation and lipid peroxidation intracellular dependent on ferrous iron or ester oxygenase.^[7]^ Previous studies suggest that ferroptosis is involved in OA progression, but the specific mechanism of action is not completely understood, and targeting ferroptosis may provide a new and effective treatment for OA.

FerrDb is a database that is collected and managed manually containing integrated information ferroptosis-related genes. This study obtained 259 FRGs from the FerrDb database, including 186 ferroptosis driver genes, 132 suppressor genes and 113 ferroptosis marker genes. In this study, we screened 282 DEGs among which 126 were upregulated and 156 were downregulated. Results of followed GO enrichment analysis shown that all DEGs mainly associated with oxidative stress, steroid hormone, and muscle cell proliferation, while results of KEGG enrichment analysis indicated that DEGs were closely related to IL-7 signaling pathway, TNF signaling pathway, HIF-1 signaling pathway, Human T-cell leukemia virus 1 infection, and NOD-like receptor signaling pathway. These findings suggested that DEGs play roles in inflammatory process in OA, suggesting possible impact of these genes’ occurrence and development of OA. We subsequentially performed Venn plot to selected the ferroptosis-related hub genes in OA with the intersection of FRGs and DEGs.

As a foremost and novel branch of artificial intelligence, machine learning is often employed to make predictions by analyzing existing data and is commonly used in the analysis of molecular biological data.^[39]^ We conducted feature selection by integrating multiple ML algorithms provided robust gene markers which may greatly improve the diagnostic performance and increase the efficiency of the test because multiple ML took more impact, factors and more complex non-liner correlation into account. By the combination of the 3 ML algorithms, we ultimately identified 4 feature genes GABARAPL1, TNFAIP3, ARNTL, and JUN for further exploration. Among the 3 ML algorithms for feature selection, SVM-REF and LASSO conducted cross validation to avoid overfitting. Followed ROC analysis of the 4 feature genes showed favorable discriminative and predictive abilities, suggesting the biological impact and potential diagnostic value of the 4 feature genes for OA. These results were validated in GSE1919 dataset. Our study also showed that GABARAPL1, TNFAIP3, and JUN was downregulated in OA synovia based on the results of RT-PCR.

It has become increasingly apparent that immunocyte infiltration plays a major role in the pathogenesis and development of OA.^[40,41]^ Arthritis foci of OA has been reported to contain abundant infiltration of CD4 + T cell. CD4 + T cell induce Th1-type immunity, which mediates with increased production of immune regulatory cell factors.^[42,43]^ Inflammation and immunity response contribute to disease progression of OA. Previous study of Rosshirt had also raised that CD16+/CD56 + natural killer cells, CD8 + T cells, CD4 + T cells, and macrophages were also highly enriched in OA joints.^[44]^ Therefore, it is necessary to gain a comprehensive understanding the immune response inside the foci of OA regarding interactions between immune cells and their microenvironment to provide new ideas and target for the clinical therapy of OA. In the present study, we observed that the levels of Effector memory CD8 T cell, Central memory CD8 T cell, Type 17 T helper cell, Type 2 T helper cell, Mast cell, Eosinophil, Activated CD4 T cell, Central memory CD4 T cell, Memory B cell, Effector memory CD4 T cell, Type 1 T helper cell, Regulatory T cell, Natural killer cell, Macrophage, MDSC, Gamma delta T cell, Immature B cell, Immature dendritic cell, CD56 bright natural killer cell, Activated CD8 T cell, and Activated B cell presented significant difference in the levels of cell infiltration between OA and normal tissue samples. We also investigated the correlation between the expression of GABARAPL1, TNFAIP3, ARNTL, and JUN and infiltrating levels of various immune cell. We found that GABARAPL1 was correlated with Central memory CD8 T cell, Mast cell, and Eosinophil; TNFAIP3 was correlated with Type 2 T helper cell, MDSC, Macrophage, Gamma delta T cell, Immature B cell, Effector memory CD4 T cell, Central memory CD8 T cell, and CD56 bright natural killer cell; ARNTL was correlated with Natural killer T cell, Monocyte, and Activated CD8 T cell. Moreover, we found that JUN was correlated with Activated CD4 T cell, Macrophage, and Natural killer T cell. From above observations, we speculated that GABARAPL1, TNFAIP3, ARNTL, and JUN affects immune responses and disease process of OA by influencing immunocytes infiltration. Further work into a complex array of immune players acting in concert behind immune response in OA is required to confirm these hypotheses.

To ascertain whether the obtained feature genes possess broader applicability, we combined our investigation with the current hotspot of immune infiltration. We extended these analyses to cancer, thereby enhancing the value of our research, and potentially broadening the applicability of these identified targets from OA to a more expansive context. By finding the intersection of immune genes obtained from InnateDB and Immport database and the 4 feature genes, we ultimately selected JUN to further explore its role in pan-cancer. We found that JUN were correlated with the clinical outcome (OS) of patients of BLCA, BRCA, CESC, CHOL, LGG, LUSC, and THYM. At the same time, we analyzed the TISIDB database and found a significant positively correlation between JUN expression level and lymphocytes, immunomodulators (including MHC molecule) and chemokines at pan-cancer level, especially in LGG. Therefore, above findings suggested that JUN had nonnegligible impact in shaping of the immune status in various cancers.

Recent evidence from many studies has shown that immunotherapy, such as anti-PD-1/L1 therapy, has emerged as the most eye-catching treatment method for malignant tumors, and the low response rate in the clinic is a great obstacle to the development of ICI therapy. In addition to PD-L1, high TMB and high MSI have been shown to be useful biomarkers for better immunotherapy response in cancer.^[33,34,45]^ Sed High TMB levels represented highly produced neoantigens, thus drive active antitumor immune responses and ultimately lead to sustained clinical response to immunotherapy. Patients with high level of MSI tend to have high level of TME, suggesting possible synergism of them and highly similar mechanism in predicting the efficacy of immunotherapy. To explore the correlation of the JUN expression with ICI response, we conducted Spearman correlation analysis, and the obtained results showed that the TMB of 11 types of cancers as well as the MSI of 8 types of cancers. These findings suggest that JUN may be a potential indicator for immunotherapy response in various cancer types.

This research has the following merits: first, integrated combination of the 3 ML algorithms for features selection increase accuracy and reduced bias to the maximum extent. Second, after preprocessing the data from different GEO datasets to eliminates discrepancies between batches, we pooled the samples from 3 datasets to expand the sample size of training dataset, promoting the statistical credibility and transferability of this study. Moreover, we validated the feature genes in another independent dataset, making our observation and conclusion sufficiently robust and reliable. There still exist a couple of limitations in the research. Applying a series of bioinformatic analyses, we identified the 4 feature genes and their abnormal expression, roles in immunity and potential biological impact in OA, while more clinical cases and experiments are needed to confirm our findings. Furthermore, the samples were relatively small even we attempt to develop enlarged database by combining the different GEO datasets and a larger sample size is required to afforded greater dependability and clarity in the results.

## 5. Conclusion

In this research, we identified 20 ferroptosis-related hub genes that expressed differentially between OA and normal control samples and ultimately selected 4 feature genes for integrating 3 ML methods. Results of ssGSEA helped reveal the heterogeneity in immune infiltration between OA and normal control samples. The correlation analysis indicated the close association between the 4 feature genes and various kinds of immune cells in OA, suggesting an underlying impact of 4 feature genes in the immunity of OA. We also analyzed the expression of JUN in pan-cancer and the correlation of JUN expression and the prognoses for patients of different cancers and results indicated that JUN might be a promising therapeutic target for pan-cancer.

## Acknowledgments

We thank the GEO datasets for providing data support to this study.

## Author contributions

**Data curation:** Duo Xia, Jing Wang.

**Formal analysis:** Duo Xia, Jing Wang, Shu Yang.

**Funding acquisition:** Jun Yao.

**Writing – original draft:** Duo Xia, Jing Wang, Shu Yang, Cancai Jiang, Jun Yao.

## Supplementary Material








