# Supplementary figures and images for: Identification of key genes and their correlation with immune infiltration in osteoarthritis using integrative bioinformatics approaches and machine-learning strategies
Source: Medicine (Baltimore). 2023 Nov 17;102(46):e35355. doi: 10.1097/MD.0000000000035355 (PMC10659738; doi:10.1097/MD.0000000000035355)

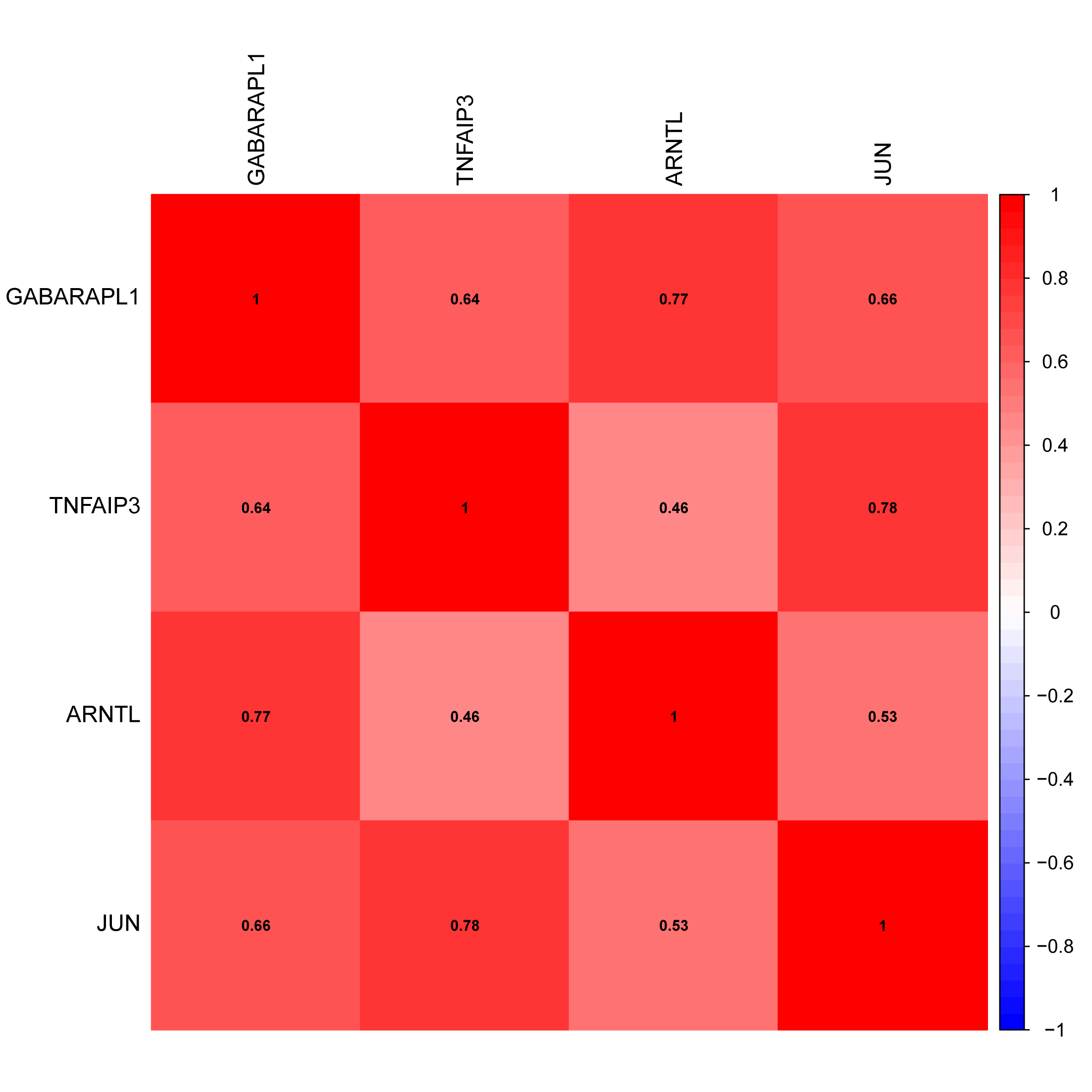

Supplement: Supplementary file 1 [file medi-102-e35355-s001.tif]

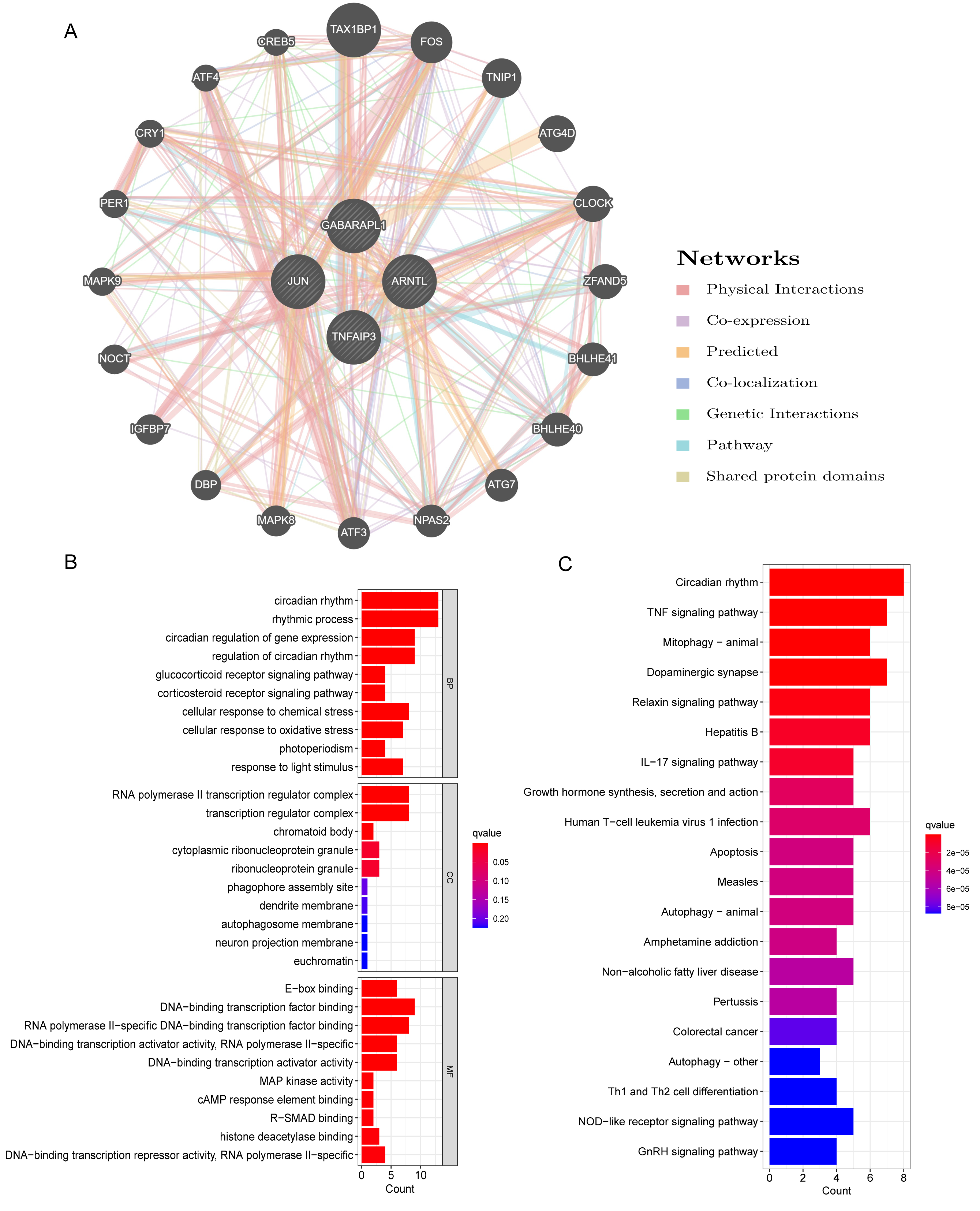

Supplement: Supplementary file 2 [file medi-102-e35355-s002.tif]

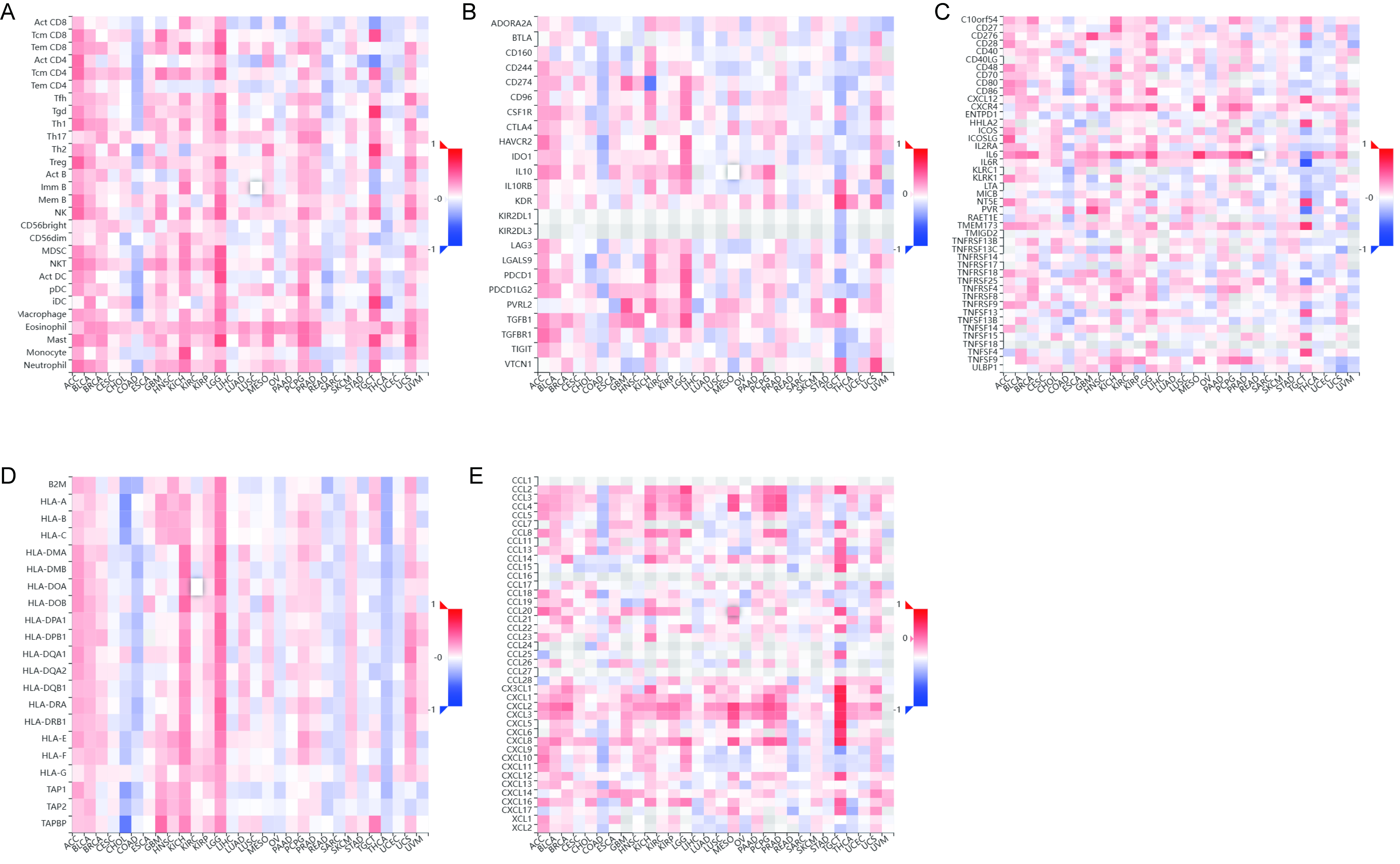

Supplement: Supplementary file 3 [file medi-102-e35355-s003.tif]

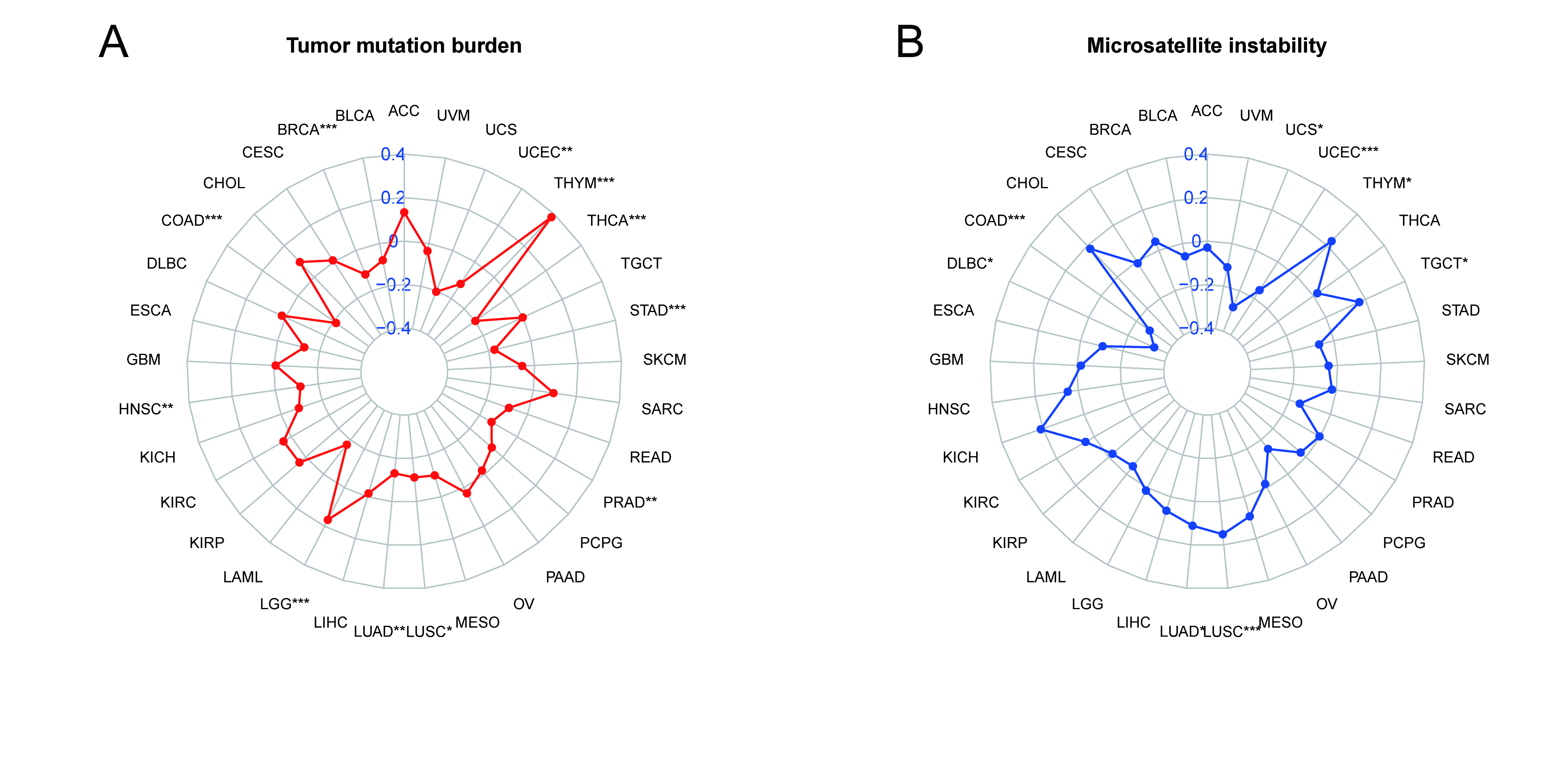

Supplement: Supplementary file 4 [file medi-102-e35355-s004.tif]
